# Inferring metabolic pathway activity levels from RNA-Seq data

**DOI:** 10.1186/s12864-016-2823-y

**Published:** 2016-08-31

**Authors:** Yvette Temate-Tiagueu, Sahar Al Seesi, Meril Mathew, Igor Mandric, Alex Rodriguez, Kayla Bean, Qiong Cheng, Olga Glebova, Ion Măndoiu, Nicole B. Lopanik, Alexander Zelikovsky

**Affiliations:** 1 Department of Computer Science, Georgia State University, 34 Peachtree St., Atlanta, 30303 GA USA; 2 Department of Biology, Georgia State University, 100 Piedmont Ave., Atlanta, 30303 GA USA; 3 Computer Science & Engineering Department, University of Connecticut, Storrs, 06269 CT USA; 4 Department of Pharmacology, University of Miami, Miami, FL USA; 5 Current address: School of Earth and Atmospheric Sciences, School of Biological Sciences, Georgia Institute of Technology, 311 Ferst Dr., Atlanta, 30332 GA USA

## Abstract

**Background:**

Assessing pathway activity levels is a plausible way to quantify metabolic differences between various conditions. This is usually inferred from microarray expression data. Wide availability of NGS technology has triggered a demand for bioinformatics tools capable of analyzing pathway activity directly from RNA-Seq data. In this paper we introduce XPathway, a set of tools that compares pathway activity analyzing mapping of contigs assembled from RNA-Seq reads to KEGG pathways. The XPathway analysis of pathway activity is based on expectation maximization and topological properties of pathway graphs.

**Results:**

XPathway tools have been applied to RNA-Seq data from the marine bryozoan *Bugula neritina* with and without its symbiotic bacterium “*Candidatus* Endobugula sertula”. We successfully identified several metabolic pathways with differential activity levels. The expression of enzymes from the identified pathways has been further validated through quantitative PCR (qPCR).

**Conclusions:**

Our results show that XPathway is able to detect and quantify the metabolic difference in two samples. The software is implemented in C, Python and shell scripting and is capable of running on Linux/Unix platforms. The source code and installation instructions are available at http://alan.cs.gsu.edu/NGS/?q=content/xpathway.

**Electronic supplementary material:**

The online version of this article (doi:10.1186/s12864-016-2823-y) contains supplementary material, which is available to authorized users.

## Background

For the past several years, RNA-Seq has revolutionized biological research through the many advantages it provides. Because of RNA-Seq, it is easier to characterize transcripts and their isoforms, to detect genes without need of prior information in the form of probes or primers, and estimate expression levels of transcripts with good precision.

In contrast to microarray data, RNA-Seq data allows frequency of expression of all transcripts without a priori knowledge of the gene sequence. RNA-Seq data can also account for all RNA transcripts producing enzymes for a given pathway. When applied to metatranscriptome data, the first challenge of pathway analysis is to decide which metabolic pathways are active in the sampled community (i.e., pathway activity detection). Recent software tools (*MEGAN4* [1] and *MetaPathways* [2] using SEED and KEGG (Kyoto Encyclopedia of Genes and Genomes) [3] annotations) enable the organization of transcripts or reads into ortholog groups and pathways by collecting all transcripts or reads represented by at least one ortholog group and providing that collection to the user. The parsimonious approach *MinPath* [4] identifies the smallest family of pathways covering all expressed ortholog groups. A more elaborate Markov Chain Monte Carlo (MCMC) approach takes into account the co-occurrences of genes in more than one pathway for analyzing metagenomic data [5]. Following pathway detection, the second major challenge of pathway analysis is to infer pathway activity levels to enable detection of differential expression. Few existing tools incorporate this step, which is the major focus of this paper.

Methods that treat pathways as simple gene sets [6, 7] are popular even though they do not use all information available. In recent years, a number of pathway analysis methods have been developed that combine knowledge of pathway topology (e.g., gene position on the pathway, gene-gene interactions, etc.) with gene expression data based on comparative analyses (reviewed in [8]). Such methods have been applied primarily to experimental studies of single organisms. There are relatively few analyses of complex metatranscriptomic datasets that incorporate pathway-level inference of metabolic activity.

The new analysis techniques presented here are suitable for investigating the underlying metabolic differences between species living in separate environments based on RNA-Seq data.

Our contribution consists of the following:

1. A novel graph-based approach to analyze pathway significance. We represent metabolic pathways as graphs that use nodes to represent biochemical compounds, with enzymes associated with edges describing biochemical reactions.
2. An implementation of an EM algorithm, in which pathways are viewed as sets of orthologs.
3. The validation of the two approaches through differential expression analysis at the transcripts and genes levels and also through real-time quantitative PCR experiments.

Pathways can also be regarded as a set of ortholog groups on which we can apply a set cover. We will use a binary ortholog group expression model to determine if there is or not RNA-Seq evidence for the expression of a given ortholog group in a given sample.

The validation step of these methods consists of extracting the genes involved in our estimated differential pathways activity levels, and analyzing their expression levels. We expect to see the differential pathway activity confirmed at the protein and contigs level. We carry this final analysis through the novel bootstrapping tool IsoDE [9].

Our experimental study was performed with RNA-Seq data from the marine bryozoan, *Bugula neritina*. Using the two novel computational approaches we implemented, we were able to find differentially expressed pathways from the data. This result has been validated by quantitative PCR (qPCR) conducted using a housekeeping gene also identified in the data. The housekeeping gene, glyceraldehyde-3-phosphate dehydrogenase (GAPDH), was chosen to normalize the qPCR data, as is standard practice. Based on our results, we applied qPCR experiments to quantify transcripts in the fatty acid elongation pathway [3].

The rest of the paper is organized as follows. Our formal models to analyze pathways and to infer pathway activity are presented in the next section entitled “Methods”. Following Methods is the Differential-analysis section in which we present how we compute differential activity between pathways. We finish by presenting and discussing our results on *Bugula neritina* data in the “Results and discussion” Section. The paper is concluded with possible future work.

## Methods

In this paper we introduce a graph-based and an expectation maximization approach to identify specific differences between biological systems on the level of ortholog groups and pathways.

**Fig. 1:**
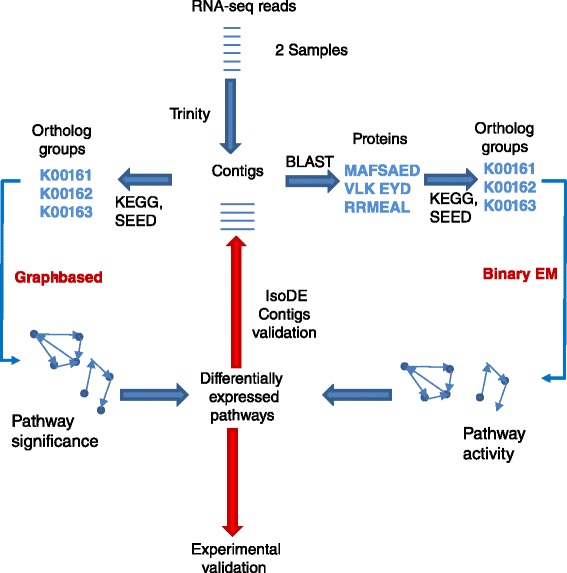
XPathway analysis flow. The branches represent the two approaches used to compute pathway significance in the case of graph-based on the left and pathway activity level in the case of the expectation maximization approach on the right. Both methods are validated by computing contigs/transcripts differential expressions and qPCR as the last step of the flow

Figure 1 presents the entire flow of XPathway tools. In the graph-based approach, we compute a *p*-value using parameters extracted from the network to answer two different statistical questions: (1) When and based on what parameter can we say that a set of proteins significantly map to a pathway? (2) What is the probability of finding such a mapping by chance given the data (transcripts/reads/proteins) and a pathway topology? Finally, significant metabolic pathways are selected by comparing the *p*-value of the original pathway with the ones from different bootstrapped samples. The expectation maximization method on the other hand uses the interaction among identified ortholog groups to infer pathway activity. The last part of the flow consists of validating both branches. First, we conduct differential expression analysis on all contigs extracted from pathways output by both branches. Secondly, a qPCR experiment is carried out on the contigs which have a fold change of 1.2 or more.

### Expectation maximization model of pathway activity

In this section we present an EM-based algorithm for inferring pathway activity levels based on metatranscriptome sequence data. Let *w* be a pathway that is considered to be a set of enzymes represented by their ortholog groups *w*={*p*_1_,…,*p*_*k*_}. Since an ortholog group can have multiple functions and participate in multiple pathways, the pathways can be viewed as a family of subsets *W* of the set of all ortholog groups *P*. Below we start by introducing a uniform binary pathway activity model based on a discrete ortholog group expressionl model.

The uniform binary pathway activity model is based on the assumptions of *uniformity*, namely that each molecule from an ortholog group participates in each active pathway with the same probability (i.e., in equal proportions) and of *binary activity*, which postulates that a pathway is active if the level of ortholog group activity exceeds a certain (possibly pathway dependent) threshold. Formally, let *δ*(*w*) be a binary variable indicating the *activity status* of *w*, i.e., *δ*(*w*)=1 if *w* is active and *δ*(*w*)=0, otherwise. Also, let the *activity level* of pathway *w* be the summation over constituent ortholog groups *g* of their participation *g*_*w*_ in *w*. Since we assume that each ortholog group *g* is equally likely to participate in each pathway containing it, it follows that $g_{w} = \left (1+ \sum _{w'\ni p, w'\ne w} \delta (w')\right)^{-1}$ and the activity level *f*_*w*_ of pathway *w* is given by

1 $$  f_{w}= \sum_{g\in w} g_{w} =\sum_{g\in w} \frac{1} {1+ \sum_{w'\ni g, w'\ne w} \delta(w')}  $$

The binary activity status of *w* is computed from its activity level *f*_*w*_ and the threshold *T*_*w*_ as follows

2 $$  \delta (w) = \left\{ \begin{array}{l l} 0 & \quad \text{if \(f_{w}<T_{w}\) }\\ 1 & \quad \text{if \(f_{w}\ge T_{w}\)} \end{array} \right.  $$

**Fig. 2:**
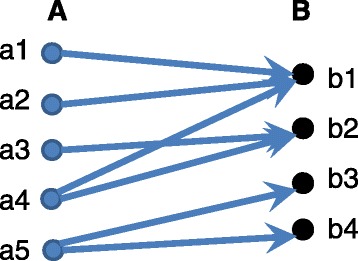
Expectation maximization approach to compute pathway activity. This bipartite graph consists of a set A representing reads/contigs/ORF/proteins and the set B is for ORFs/proteins/ortholog groups/EC (Enzyme Commission) numbers. The arcs represent mapping between elements of both sets. For our binary EM, the set A consists of contigs mapped to ortholog groups and the weight of each arc is 1

The uniform binary model described by Eqs. (1)–(2) can be solved using a simple iterative algorithm. The algorithm starts with assigning activity status *δ*(*w*)=1 to each pathway *w*∈*W*, i.e., *Δ*^0^(*W*)={*δ*^0^(*w*)|*w*∈*W*}←1 and then repeatedly updates the activity level according to (1) and the activity status according to (2). The procedure terminates when the status sequence *Δ*^0^(*W*)=1,*Δ*^1^(*W*),*Δ*^2^(*W*),… starts to oscillate *Δ*^*n*+*k*^(*W*)=*Δ*^*n*^(*W*) or converges. In all our preliminary experiments, an oscillation with period *k*=2 is achieved in at most 10 iterations. Also the threshold *T*_*w*_ does not significantly change the order of pathways sorted with respect to their activity levels estimated as the mean *f*_*w*_ after convergence. The model is represented in Fig. 2.

Although the uniform binary model allows the computation of pathway activity by assigning ortholog groups to pathways, it does have some limitations hindering it for capturing specific attributes of the metabolic network. For example, the binary uniform model assigns only value 1 or 0, if the ortholog group belongs to a pathway or not, respectively. This yes or no assumption is not always true since there may be a fractional part of an ortholog group belonging to different pathways. Moreover, the uniformity model is not easily applicable to natural processes because all assignments are never equally likely. Finally the model is not completely stable but rather periodic with some subsets of ortholog groups fluctuating between pathways.

### Graph-based estimation of pathway significance

Ideally, a comprehensive pathway analysis method would take into consideration the position and role of each gene in a pathway, the efficiency with which a certain reaction is carried out, and some limiting factors (e.g. dealing with metagenomics data or not). With genome data, it is possible to consider pathways size, gene length and overlap in gene content among pathways [5] to compute the relative abundance of pathways and pathway ranking, but this approach might not work with RNA-Seq data especially in the absence of a genome reference.

Henceforth, in our second approach, each pathway is viewed as a network of enzymes also called EC numbers (Enzyme Commission numbers) in order to compute their statistical significance. Significance of pathway activity in a sample is measured by the randomness of the positions of matched enzymes in the corresponding KEGG pathway graph. The randomness is measured using a permutation model for finding significant pathway alignments and motifs [10].

This model assumes that the subset of expressed enzymes in an active annotated pathway should be connected. The enzyme permutation model finds the average vertex degree in the subgraph induced by expressed enzymes. Then the same parameter is computed for sufficiently many random permutations of enzyme labels. The statistically significant match should have density higher than 95 % of permutations. Specific characteristics of the graph taken into account in our analysis are:

1. Number of nodes. A node represents a protein that got mapped during BLAST. KEGG usually assigns a green color to those proteins in the graph.
2. Density = (Number of edges)/(Number of nodes − 1)
3. Fraction of 0 in and out-degree nodes. Let call this number *x*. *x* is defined by:
  x = ((number of nodes with out-degree = 0) + (number of nodes with in-degree = 0)) / 2 * (number of nodes)
4. We also consider other criteria such as (1) number of green connected components, (2) Largest number of nodes in a connected component and (3) Largest number of edges in a connected component.

Using these metrics, we compute the density of the induced graph composed of only mapped proteins. We obtain the names of those proteins through EC numbers on the graph. Below, we present two graph-based models, the vertex label swapping and the edge swapping for random graph generation, to analyze pathways. This model is explained by the left side of Fig. 1.

### Model 1: Vertex label swapping for random graph generation

**Fig. 3:**
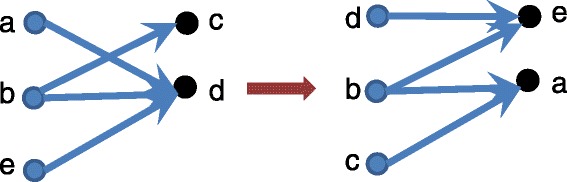
Vertex labels swapping model for random graph generation. We only swap vertices which have different labels. A label is an attribute of a vertex representing a mapped or not protein



In this model, we keep the same topology but we allow swapping of labels between two vertices (Fig. 3 presents an example). One known issue of this approach is that vertices with high degree always get connected. This might lead to too many significant matches, thus increasing the false positive rate. The vertex label swapping algorithm can be represented as follows:

### Model 2: Edge swapping for random graph generation

**Fig. 4:**
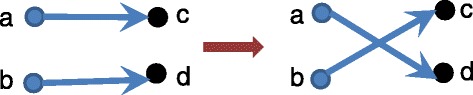
Edge swapping model for random graph generation. Before swapping the edges, we check that the in and out-degree of the vertices involved remain the same

Because of the bias in the vertex label swapping model, we also implemented edge swapping. Here, the idea is to keep the in-degree and out-degree of each node the same, swapping nodes only if these values do not change. We keep vertex labels the same. Figure 4 presents an example when we permute two edges.

The edge swapping algorithm can be represented as follows:



### Differential analysis of pathway activity and significance

#### Differential analysis of pathway activity

The goal of this analysis is to determine which pathway needs to be considered more closely to understand the difference in the metabolism of two organisms. For this purpose, we use the pathway expression computed from the binary model presented earlier. First we compute expression of each pathway present in the set of pathways we get from KEGG for a given sample. Then we compute the difference between the expression of each pathway. Under this model, the pathways selected as having differential activity are the ones where the ratio of their expression is greater than a certain threshold. Table 3 presents our results on differential analysis of pathway activity.

#### Differential analysis of pathway significance

Differential analysis of pathway significance is based on the *p*-*value* described in the graph-based sub-section of Methods. We randomly permute each pathway graph generating *m* different graphs. Note that even the smallest pathway graphs contains at least 15 nodes and about 40 edges which is sufficient to generate default *m*=200 distinct random graphs. A pathway is significant if the *p*-*value* of the mapping is less than 5 %. The *p*-*value* is the position of the original graph when placed in the sorted list of all randomly generated graphs sorted first by “density” (largest to smallest) and then by the number of nodes having 0 in-degree or 0 out-degree (smallest to largest). A pathway is *significant* if its *p*-*value* is less than 5 %, *very significant* if its *p*-*value* is less than 1 % and *the most significant* if its *p*-*value* is less or equal to 0.5 %.

Let *p*1 be the *p*-*value* for pathway *X* in sample 1 and let *p*2 be the *p*-*value* for pathway *X* in sample 2. We say that pathway *X* is differentially significant between the two samples if the probability computed by the equation of *probDiff*(*X*) below is greater than 50 %.

$$ probDiff(X) =\left\{ \begin{array}{ll} (1-p1)*p2 & \text{if~} p2 \geq p1 \\ (1-p2)*p1 & \text{if~} p2 < p1 \end{array} \right. $$

**Fig. 5:**
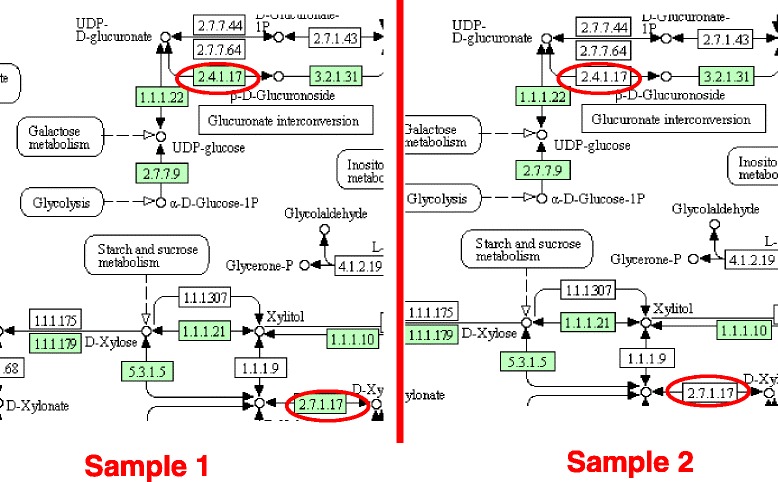
Pathway differential analysis. In sample 1, the mapped enzymes (filled rectangles) form a sub-graph with density = 1.475, the number of 0 in/out degree = 0.11 and *p*-value = 0.5. In Sample 2, the mapped enzymes (filled rectangles) form a sub-graph with density = 1.375, the number of 0 in/out degree = 0.22 and *p*-value =.74. Based on these *p*-value, we say that this pathways is differentially significant

For example, let us consider *m*=200 randomly generated graphs and the vertex label swapping model. In Fig. 5 representing part of the Fatty acid elongation pathway (ko00062), the mapped enzymes (filled rectangles) in sample 1 form a sub-graph with density = 1.875 and the number of 0 in/out degree = 0.11 for that sub-graph. After sorting the graph, the position of our original graph is the first, hence *p*-value *p*1=0.5 *%* (most significant pathway given the 200 graphs). In Sample 2, the mapped enzymes (filled rectangles) form a sub-graph with density = 1.375, number of 0 in/out degree = 0.22 for that sub-graph and its position after sorting is 148. This results in a *p*-value *p*2=74.5 *%* (not a significant mapping).

Based on the value of p1 and p2, *probDiff*(*ko*00062)=.74 which is greater than 50 %. We conclude that ko00062 is differentially significant in the two samples.

## Results and discussion

### Data preparation

*Bugula neritina* is a colonial marine invertebrate found in temperate waters around the world [11]. *B. neritina* associates with an uncultured microbial symbiont, “*Candidatus* Endobugula sertula” [12] that has been shown to produce bryostatins, bioactive compounds that protect the host larvae from predation [13, 14]. Because of the pharmaceutical potential of the bryostatins, and the inablilty to grow the symbiont in the laboratory, we chose to examine host gene expression in the presence and absence of the symbiont to understand more about the molecular underpinnings of this relationship. In addition, as the symbiont endows the larvae with high concentrations of bryostatins compared to the adult [15, 16], we also wanted to examine host gene expression in portions of the colony that possess reproductive structures termed ovicells, and those without ovicells.

Adult colonies of *B. neritina* growing on floating docks were collected from four locations on the Eastern coast of the USA: Radio Island Marina and Yacht Basin Marina, Morehead City (North Carolina) in March 2012, Oyster public docks, Oyster (Virginia) in June 2012, and Indian River Inlet, Delaware City (Delaware) in June 2012. The colonies were rinsed in filtered sea water and preserved in TRIzol reagent (Invitrogen, Carlsbad, CA) at −80 degree celsius prior to RNA extraction. Total RNA was then extracted from the preserved samples (RNeasy Mini kit, Qiagen,Inc., Valencia, CA, USA). The RNA was purified and treated with RNase-free DNaseI to remove any contaminating DNA. The purified total RNA was processed according to standard operating procedure for preparation of mRNA library for sequencing (TruSeq RNA Sample Preparation Kit, Illumina, San Diego, CA, USA). The adapter-ligated cDNA library was hybridized to the surface of Illumina flow cell and sequenced on an Illumina HiSeq 2500 sequencing platform. The paired-end reads were assembled de novo using Trinity software package [17] and the assembled contigs were annotated by performing blastx searches (Translated Query-Protein Subject BLAST 2.2.26+) against the Swiss-Prot database.

The samples used for analysis include: Lane 1: symbiotic; Lane 2: non symbiotic (aposymbiotic) [18]; Lane 3: symbiotic, with ovicells; and Lane 4: symbiotic, without ovicells. The symbiotic relationship was assessed in the collected colonies by PCR analysis for the presence of the bryostatin biosynthetic gene cluster gene, bryS [18, 19]. The reads were assembled into contigs by Trinity. We conducted a Trinity assembly on the union of all reads - about 221,818,850 2 ×50 bp reads in total. We obtained 166,951 contigs, after filtering with RSEM-isopct-cutoff=1.00, also 76,769 ORFs, 37,026 BLAST hits of translated ORFs against the SwissProt database and around 12,748 proteins hits. This translates to 59.37 % ORFs hits and 63.35 % contigs hits. Using IsoDE, we were able to identify 1485 differential expressed genes between the two different conditions, the symbiotic and aposymbiotic *B. neritina*.

### Pathway extraction and graph generation

By *de novo* co-assembly of RNA-Seq data and BLAST-ing resulting contigs against protein databases, with a certain confidence, we can infer the ortholog groups expressed in the sample. This is an important attribute of KEGG. We use KEGG to generate pathways from Trinity contigs and proteins. From the pathway databases we can easily extract the enzyme information associated with each pathway. We actually extracted all pathways along with all mapped proteins.

KEGG represents proteins as KO numbers and we also follow this representation. It uses KGML, an exchange format of KEGG pathway maps, to interact with external applications. The next step was to download all KGML files associated with the pathways using the API provided by KEGG. To convert KGML files to graph of node and vertices, we implemented and ran a novel tool called KGMLPathway2Graph. Mapping the output of KGMLPathway2Graph with KO numbers from KEGG analysis of our data, allowed us to compute patways with significant *p*-*values*.

### Results

Pathway expression differences in symbiotic and aposymbiotic *Bugula neritina* (Lanes 1 and 2) are shown in Table 1 and in Table 2 respectively following vertex labels permutation and the edges permutation models. We ran our graph-based models Algorithm 1 with *m*=200 graphs each generated after *n*=1000000 permutations of labels or edges. This high number of permutations is necessary to introduce sufficient differences in the generated graphs. Table 3 presents the results on differential analysis of pathway activities between the same two *Bugula neritina* conditions.

**Table 1:** Vertex label permutation: the *p*-values of pathways are computed from the symbiotic (Lane 1) and aposymbiotic (Lane 2) *B. neritina* data. This table presents the most significant divergence in pathway results, using the criteria described in the “Methods” Section, they are declared differentially significant

Pathway	*p*-value from	*p*-value from	ProbDiff
	symbiotic Bugula	aposymbiotic Bugula	
ko04146	.99	.05	.94
ko03008	.99	.05	.94
ko03013	.99	.05	.94
ko00983	.99	.05	.94
ko04530	.99	.05	.94
ko00062	.01	.75	.74
ko00400	.01	.99	.98
ko00071	.99	.01	.98
ko00100	.99	.01	.98
ko00910	.04	.99	.95
ko04122	.99	.03	.97
ko04713	.99	.01	.99

**Table 2:** Edge permutation: the *p*-values of pathways are computed from the symbiotic (Lane 1) and aposymbiotic (Lane 2) Bugula data. This table presents the most significant divergence in pathway results, using the criteria described in the “Methods” Section, they are declared differentially significant

Pathway	*p*-value from	*p*-value from	ProbDiff
	symbiotic Bugula	aposymbiotic Bugula	
ko04146	.99	.05	.94
ko03008	.99	.05	.94
ko03013	.99	.05	.94
ko00983	.99	.05	.94
ko04530	.99	.05	.94
ko00400	.01	.99	.98
ko04122	.99	.03	.97
ko04713	.99	.01	.99
ko00130	.01	.75	.74
ko00120	.01	.99	.98
ko00072	.99	.01	.98
ko00120	.99	.01	.98
ko00230	.04	.99	.95
ko00627	.99	.03	.97
ko00770	.99	.01	.99
ko00980	.99	.03	.97
ko04630	.99	.01	.99

**Table 3:** Pathway activities levels with ratio

Pathway	Expression	Expression	Ratio between
	from symbiotic	from aposymbiotic	pathway
	Bugula	Bugula	expressions
ko00300	2.75	0.38	7.27
ko00290	4.26	1.71	2.49
ko04712	1.77	0.77	2.30
ko00903	1.88	0.84	2.25
ko01220	2.24	1.10	2.04
ko00981	2.00	1.00	2.00
ko04744	3.55	1.82	1.95
ko00626	1.48	0.76	1.93
ko00624	1.17	0.67	1.76
ko00072	2.17	1.25	1.74
ko00730	2.50	1.50	1.67
ko04730	3.80	2.40	1.58
ko00363	2.92	1.88	1.56
ko05150	2.13	1.38	1.55
ko04112	3.00	2.00	1.50
ko05219	1.67	2.50	0.66
ko00625	1.00	1.98	0.50
ko00984	1.00	2.00	0.50
ko00592	1.25	3.02	0.42
ko00965	0.66	1.66	0.40
ko00940	1.42	3.71	0.38
ko00460	0.60	2.02	0.30
ko00944	0.17	1.17	0.14

Expression represents the expression level of the pathway activity in symbiotic (Lane 1) and aposymbiotic (Lane 2) *B. neritina* data. This table presents pathways with a ratio of 1.5 or higher in their activity level or pathways with a ratio of 0.66 or lower from the opposite direction. Using the criteria described in section 2, they are found to significantly differ in activities level

In Additional file 1, we present a summary of transcripts differential expression (DE) analysis results using IsoDE [9] and pathway activity inference results.

### Validation

From our statistical analysis, we identified some pathways that were differentially expressed (DE) by all methods. The next step was to experimentally validate these results. The first validation step is done through IsoDE, a software to analyze differentially expressed genes. Through KEGG, we are able to get all the proteins (contigs) participating in a pathway. IsoDE then indicates which of those contigs are also DE. From those DE contigs, we extracted the genes to be tested via quantitative PCR (qPCR), the next validation step.

The goal of qPCR is to quantify the level of expression in the symbiotic and aposymbiotic *B. neritina*. It is used to validate the gene expression given by IsoDE. Primers were designed using Primer 3 Plus [20]. Total RNA from recently collected symbiotic and aposymbiotic *B. neritina* colonies was converted to cDNA using Superscript III (Invitrogen, Carlsbad, CA, USA) using random hexamers according to the manufacturer’s instructions. cDNA was subjected to qPCR analysis after and expression in the samples was compared using the *ΔΔ*Ct method [21]. The glyceraldehyde-3-phosphate dehydrogenase gene, a housekeeping gene identified from the *B. neritina* transcriptome (contig m.4423) was used for normalization [22, 23]. The expression of three genes identified as being differentially expressed in symbiotic and aposymbiotic animals from the fatty acid elongation pathway (ko00062) were compared.

Using IsoDE, nine gene pathways were chosen from KEGG and 2485 top differentially expressed contigs were taken from the list of all contigs. Within the selected pathways, there was a total of 637 contigs extracted. Each gene in these contigs was checked for fold change 1.2 or higher. Next, the number of genes that had significant fold change was compared to the total number of genes in the pathway. The fatty acid elongation pathway (ko00062) was chosen for further investigation by qPCR [3].

The fatty acid elongation pathway contains fourteen (14) KEGG mapped contigs and three (3) of those were found significantly differentially expressed. They are very-long-chain 3-oxoacyl-CoA reductase (DHB12), 3-ketoacyl-CoA thiolase (fadA) and 3-ketoacyl-CoA thiolase B, peroxisomal (THIKB). Once all the contigs in the pathway were checked, additional information was compiled: KEGG pathway and protein numbers, contig number, UniProt accession number and predicted fold change between symbiotic and aposymbiotic *B. neritina* [24]. qPCR primers were designed using Primer 3 Plus and ordered from Integrated DNA Technology [20]. The primers were tested using cDNA at concentrations from 1 *ng*/*μL* to 0.1 *pg*/*μL*. In order to use △△Ct method, every primer had to have the same efficiency and efficiency around 100 % [21].

RNA was extracted from symbiotic and aposymbiotic *B. neritina* colonies. Following the Direct-zol RNA MiniPrep protocol (Zymo Research Corp., Irvine, California, USA), 50 mg of *B. neritina* tissue was homogenized and RNA was extracted. Then the RNA was further purified using the OneStep PCR Inhibitor Removal Kit (Zymo Research Corp., Irvine, California, USA). To eliminate any contaminating genomic DNA, a DNase I treatment was performed according to the manufacturer’s protocol (DNase I Recombinant, RNase-free, Roche, Mannheim, Germany). Finally, the RNA was further purified with the RNA Clean-up and Concentrator Kit (Zymo Research Corp., Irvine, California, USA). The concentration of RNA was quantified in triplicate using a Nanodrop spectrophotometer.

Both symbiotic and aposymbiotic cDNA were synthesized using the Superscript III protocol (Superscript III First Strand Synthesis System for RT-PCR, Invitrogen by Life Technologies, Carlsbad, California, USA) with random hexamers. qPCR primer efficiencies were determined using qPCR. All qPCR reactions were performed using 7500 Fast Real-Time PCR system (Applied Biosystems) with hot-start Taq polymerase, SYBR Green fluorescent dye and ROX passive reference dye (Maxima SYBR Green/ROX qPCR Master Mix (2X), Life Technologies, Carlsbad, California, USA). The efficiencies for each primer pair were calculated using the slope of amplification curve in the equation E = 10(-1/slope) [25].

The expression levels of three genes, *fadA*, DHB12, and THIKB were measured in symbiotic and aposymbiotic *B. neritina* with G3P acting as an endogenous control. The reactions were run in triplicate for symbiotic and aposymbiotic cDNA along with a negative template control. The Ct averages and standard deviations were calculated to find the Ct differences between the target gene and the control (△Ct) and △Ct standard deviation. △△Ct was calculated by subtracting the symbiotic or aposymbiotic △Ct by the symbiotic △Ct. This resulted in symbiotic △△Ct equal to 0 to compared the fold change between symbiotic and aposymbiotic expression levels.

As presented in Table 4, the fold change predicted by differential expression analysis, using IsoDE, for these three genes indicated that expression was higher in the aposymbiotic B. neritina. *fadA* had a predicted fold change of 2.91, while DHB12 had a value of 1.90 (non-significant difference), and THIKB equaled 2.84. qPCR analysis showed that when aposymbiotic gene expression was compared to symbiotic gene expression, *fadA* had 6.88 higher expression in aposymbiotic *B. neritina*. DHB12 had 0.66 times lower expression and THIKB had 2.52 higher expression, indicating that computational method closely predicted expression in independent biological samples.

**Table 4:** Experimental quantification of fatty acid elongation gene expression by qPCR in symbiotic and naturally aposymbiotic *B. neritina*

Genes	fadA	DHB12	THIK
Fold change of gene expression in aposymbiotic *B. neritina* compared to symbiotic by FPKM analysis	2.91	1.90	2.84
Gene expression in symbiotic*B.* *neritina* by qPCR analysis	2.46	4.34	4.34
Gene expression in aposymbiotic*B.* *neritina* by qPCR analysis	29.32	2.85	10.95
Fold change of gene expression in aposymbiotic *B. neritina* compared to symbiotic by qPCR analysis	6.88	0.66	2.52

### Discussion

Although the EM and the graph-based methods worked on the same data generated by KEGG, the input to each approach were very different. For example, the Trinity output of sample1 on KEGG generates about 306 pathways. All of these pathways were considered for EM methods while only a small subset of 80 was used as input to each of the graph-based model. Different factors contributed to this reduced number of pathways analyzed in the edge/vertex swapping model: (1) We were not able to extract the KGML of all pathways from from KEGG; (2) We were not able to convert all KGML to actual graphs and (3) Some graphs did not carry enough mapping to be significant (we excluded pathways with less than 3 ortholog groups mapped).

Consequently, the graph-based approaches yield considerable fewer differentially expressed pathways than EM methods although results from both models in the graph-based approaches were very consistent. Also, the graph-based analysis appears to be more stringent selecting only the pathways which are the farthest apart according to our statistic criteria.

Looking at the overall small number of differential pathways, we can say that *B. neritina* with or without the symbiotic relationship still exhibits very similar metabolic reactions. As shown in Table 5, the over all difference in the pathway activity and differentially expressed transcripts between the two samples is very small.

**Table 5:** Percentage of differentially expressed contigs with fold change (FC) of 2 and 1.5 respectively

	FC = 2	FC = 1.5
Pathway	8 %	12 %
Contigs	13 %	28 %

Because working with non-model organisms, such as *B. neritina*, is more challenging due to the lack of tools for genetic manipulation, for future work, we plan on the following. First, run XPathways tools in other organisms, including model organisms to further verify their efficacy and second, extend the model to handle not only metabolic pathways put also signaling pathways.

## Conclusions

XPathway tools are able to efficiently infer pathway activity as well as pathway significance, using an expectation maximization and a graph-based approach, respectively. Rather than trying directly to identify differentially expressed genes from RNA-Seq data for a non-model organism, XPathway tools allows to more accurately predict differential expression of genes using the wealth of information collected in the KEGG database for related organisms. Our experimental comparisons on *Bugula neritina* RNA-Seq data with or without the symbiotic bacteria enabled the identification of metabolic pathways with differential activity. The qPCR experiment successfully validated our findings.

## Additional file

Additional file 1 Supplementary table. The Supplementary table is supplied in PDF format. (PDF 16 kb)
