# Supplementary material for: Inferring metabolic pathway activity levels from RNA-Seq data
Source: BMC Genomics. 2016 Aug 31;17(Suppl 5):542. doi: 10.1186/s12864-016-2823-y (PMC5009525; doi:10.1186/s12864-016-2823-y)
Supplement: Additional file 1 — Supplementary table. The Supplementary table is supplied in PDF format. (PDF 16 kb) [file 12864_2016_2823_MOESM1_ESM.pdf]

Supplementary table

Table S1 presents a summary of selection of genes for qPCR experiment. only pathway with a ratio of DE transcript of 15% or more were selected.

Pathway	Mapped-contigs	DE-contigs	Ratio-of-DE
ko00062:Fatty acid elongation	14	3	21.43%
ko00100:Steroid biosynthesis	8	1	12.50%
ko00250:Alanine, aspartate and glutamate metabolism	39	4	10.26%
ko04146:Peroxisome	98	15	15.31%
ko03008: Ribosome biogenesis in eukaryotes	67	10	14.93%
ko03013: RNA transport	148	22	14.86%
ko00983:Drug metabolism - other enzymes	28	4	14.29%
ko04530:Tight junction	237	15	6.33%

Table S1: DE contigs from selected pathways.
